# Role of Medio-Dorsal Frontal and Posterior Parietal Neurons during Auditory Detection Performance in Rats

**DOI:** 10.1371/journal.pone.0114064

**Published:** 2014-12-05

**Authors:** Kaitlin S. Bohon, Michael C. Wiest

**Affiliations:** Wellesley College Neuroscience Program, Wellesley, Massachusetts, United States of America; UNLV, United States of America

## Abstract

To further characterize the role of frontal and parietal cortices in rat cognition, we recorded action potentials simultaneously from multiple sites in the medio-dorsal frontal cortex and posterior parietal cortex of rats while they performed a two-choice auditory detection task. We quantified neural correlates of task performance, including response movements, perception of a target tone, and the differentiation between stimuli with distinct features (different pitches or durations). A minority of units—15% in frontal cortex, 23% in parietal cortex—significantly distinguished hit trials (successful detections, response movement to the right) from correct rejection trials (correct leftward response to the absence of the target tone). Estimating the contribution of movement-related activity to these responses suggested that more than half of these units were likely signaling correct perception of the auditory target, rather than merely movement direction. In addition, we found a smaller and mostly not overlapping population of units that differentiated stimuli based on task-irrelevant details. The detection-related spiking responses we observed suggest that correlates of perception in the rat are sparsely represented among neurons in the rat's frontal-parietal network, without being concentrated preferentially in frontal or parietal areas.

## Introduction

Imaging studies in humans support the existence of a frontal-parietal network that subserves attention to external stimuli in multiple stimulus modalities [1], [2] as well as conscious perception [3]. Specifically prefrontal [4], dorsal supplementary motor [5] and premotor [6] frontal areas have been implicated in attentional performance and functional coupling with posterior parietal cortex (PPC) areas. In terms of perception, imaging studies suggest that dorsal prefrontal and posterior parietal areas contribute to conscious visual perception [7]. Synchronization of gamma oscillations between prefrontal and parietal sites has been reported as a correlate of conscious visual perception [8], [9], again suggesting the two areas function together as an integrated network during perception.

In addition to its role in movement planning and preparation [10], in rats, medio-dorsal frontal cortex (MDFC) has been reported to participate in a variety of executive functions [11] including working memory [12] and value-based decision making [13]. Although intriguing functional similarities between primate and rat medial frontal cortices have been noted [14], it remains unclear whether rodents possess a true homologue of primates' granular frontal cortex [15]. The MDFC is also required for inhibiting a behavioral response until a trigger stimulus [16], but little is known about its involvement in auditory processing [17]. Similarly, rat posterior parietal cortex (PPC) receives multi-modal sensory inputs including auditory inputs [18], [19] and participates in sensory working memory and decision-making [20], but few studies have addressed its auditory functions [21].

To test whether the rat MDFC and PPC are involved in auditory perception we used multi-electrode arrays to record multi-unit spiking activity from MDFC and PPC neurons in rats performing a two-choice auditory detection task. In some of the behavioral detection sessions two distinct (in terms of pitch or duration) but equally rewarded target tones were presented, to characterize the fraction of units in each area that carried information about task-irrelevant sensory features of the target tone.

We targeted our parietal arrays to a posterior parietal cortical area previously shown to exhibit perceptual responses during a visual detection task [22], and known to have anatomical connections to the MDFC [18], from which we recorded simultaneously. Our elongated frontal arrays (2×16) sampled regions of medio-dorsal frontal cortex, including prefrontal cortex where basal forebrain cholinergic amplifications of visual and tactile responses have been reported [23], and secondary motor cortex, because analogous (dorsal premotor and prefrontal) areas in monkeys show tactile perceptual responses [24] and task-related coherence with parietal areas [6], [25].

We applied a choice probability analysis to identify potentially perception-related spiking rate modulations. Those units that could significantly discriminate successful reports of stimulus presence from correct reports of stimulus absence were considered “candidate perceptual units” (CPUs). Although consistent with perceptual responding, these units could well be signaling the distinct left and rightward movements made by the animals to report their perception, rather than the perception itself. We therefore applied a further analysis to identify those CPUs whose responding was consistent with purely direction-related information on correct and incorrect trials, independent of the animals' perception. This left a substantial fraction of units whose responding was not consistent with signaling only movement direction; these latter response modulations were likely perception-related.

## Methods

### Animals

Neurophysiological data were obtained from five male Long-Evans rats, *Rattus norvegicus* (500–700 g, Charles River Laboratories, Wilmington, MA). The animals were housed in pairs (before surgery) or individually (after surgery) on a 12∶12 light/dark schedule (lights on at 6 am/off at 6 pm). Training took place once per weekday during either a morning (10 am) or afternoon (1:30 pm) session. Rats consumed 2–10 mL of water during typical sessions. Approximately one hour after training, rats received free access to water for 15–20 minutes, during which they typically consumed about 15 mL of water. Rats were allowed free access to water over the weekend. All rats were weighed daily during water scheduling to ensure that body weight did not drop below 85% of *ad libitum* weight, as measured after 48 hours of free access to water. If this happened, rats temporarily ceased training and were given free access to water until their weight reached their *ad libitum* weight the previous Sunday. All rats were allowed food *ad libitum*.

### Surgery

Rats were removed from water scheduling at least three days before electrode array implantation surgery. For implantation surgery, rats were anesthetized with isoflurane in a stereotaxic apparatus (1–2% in O_2_). Chronic 32-microelectrode arrays (Innovative Neurophysiology, Inc.) were implanted in right frontal (2.0 mm anterior to bregma, 0.75 mm right of midline and 1.5 mm beneath the brain surface) and right parietal cortex (4.15 mm posterior to bregma, 3.5 mm right of midline and 1.2 mm beneath the brain surface). The frontal array was a 2×16 grid and the parietal array was a 4×8 grid, both with an inter-electrode spacing of 150 µm and row spacing of 300 µm. Arrays were fixed in place with dental cement. Three of the five rat brains we recorded from in this study were recovered for histological processing. Of these animals parietal array locations were verified in all three, but frontal tracks were only verified in one of the three. Therefore our frontal recordings may have been more superficial than our target depth. After surgery, rats were allowed to recover for one week with *ad libitum* access to food and water. Rats were weighed and assessed for signs of pain daily for one week following surgery.

All procedures involving animals were approved by the Wellesley College Institutional Animal Care and Use Committee, in accordance with the guidelines set by the American Association for Accreditation of Laboratory Animal Care (AAALAC) International.

### Two-choice auditory detection task

**Figure 1:**
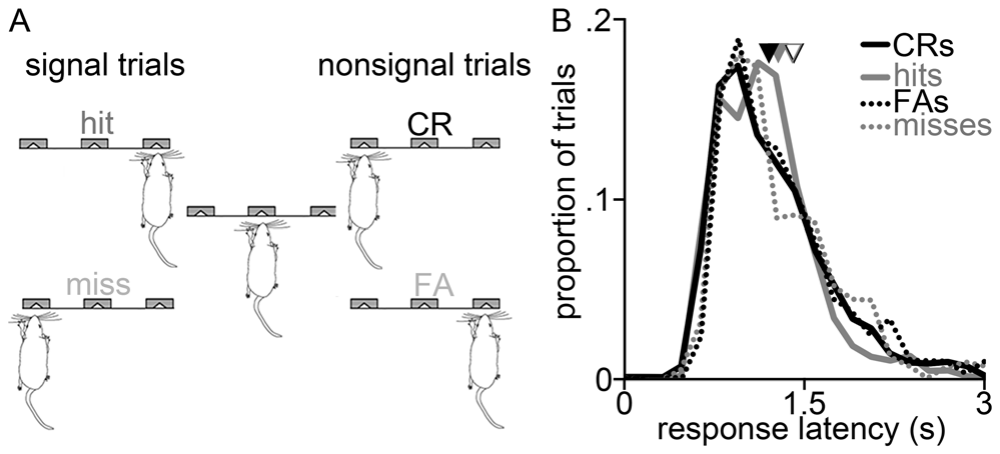
Two choice auditory detection task. (**A**) A trial is initiated by the rat's nosepoke (*center*), at which time the signal tone is either presented (*right*) or not presented (*left*). Licking at the right lickometer after signal presentation is a hit rewarded by water; failing to lick is a miss. On non-signal trials, licking to the left is a correct rejection (CR) rewarded by water; licking to the right is a false alarm (FA). (**B**) Hit (*gray solid*), CR (*black solid*), FA (*black dashed*) and misses (*gray dashed*) response latency histograms. Arrowheads indicate the average response latency for hits (*solid gray*), CRs (*solid black*), FAs (*open black*) and misses (*open gray*).

Behavioral training and neural recording took place in a standard operant chamber (80003NS, Lafayette Instrument). Rats initiated a trial by poking their nose into a cone (nosepoke). On signal trials, a fixed period of time following the nosepoke (either 10 ms, 400 ms or 500 ms), a tone would play. The delay prior to stimulus onset was fixed within a session, and rats were not required to maintain their nosepoke during the delay. On nonsignal trials, no tone was played. For 5.5 seconds following the nosepoke, rats were able to obtain a water reward at a lickometer to the right of the nosepoke following a signal, and at lickometer to the left of the nosepoke following lack of a signal (Figure 1A). Signal trials during which the rat received water were classified as *hit trials*, while signal trials during which the rat did not receive water were classified as *miss trials.* Nonsignal trials during which the rat received water were classified as *correct rejection (CR) trials*, while nonsignal trials during which the rat did not receive water were classified as *false alarm (FA) trials.* A new trial could be initiated immediately after the reward period of a correct trial. Incorrect (miss or FA) trials were followed by a lights-out penalty period of 15 seconds during which a trial could not be initiated. The median inter-trial interval (ITI), measured as the time between the first response lick and next nosepoke, was 10 SD 7 seconds. Reward for CRs (left lickometer) and hits (right lickometer) was identical, resulting in a total of about 2 mL of water being consumed during a typical session. During standard two choice tasks, all signals were 4000 Hz, 75 dB SPL, 500 ms. During two-stimuli two choice tasks, signals could either differ in pitch or in duration. For two pitch sessions, low pitch tones were 2500 Hz and high pitch tones were either 4000 Hz or 3000 Hz, with equal intensities of 75 dB SPL. For two duration sessions, long tones lasted 500 ms and brief tones 50 ms, with equal pitches. Rats were trained to respond to all tones uniformly, regardless of pitch or duration. Training or recording sessions typically lasted 30–80 minutes.

### Electrophysiological recordings

Multi-unit action potentials were recorded with a Cerebus Data Acquisition System (Blackrock Microsystems). Head-stage amplifiers were Triangle Biosystems International M62 (input impedance 50 MΩ @ 1 kHz). All spikes were sorted manually online using spike-sorting software (Blackrock Microsystems). In an effort to count each unit towards our population percentages only once despite the potential stability of units across days on chronically implanted arrays (Dickey et al. 2009), we performed an automated firing rate-based exclusion procedure. We compared the baseline firing rate, as measured by the action potential count in the second prior to trial initiation, of units recorded from the same electrode on consecutive days. If common-electrode units were found to have average firing rates within one standard deviation on consecutive days, the unit was removed from the later day's analysis. This exclusion found that approximately 60% of units had similar firing rates on consecutive days. The units on consecutive days with similar average rates could have reflected activity from different neurons, but in our *reduced data set* we conservatively treat these units as repeated samples of the previous days' units, and omit them.

### Choice probability analysis

To quantify how well multi-unit spiking data could predict the animal's behavior (or different trial types) on individual trials, we applied a receiver operating characteristic (ROC) analysis. This method gives a “choice probability” (CP) index between 0 and 1 which measures how well an ideal observer could predict the animal's behavior, given only the spike count from a specified time window during each trial. Choice probability allows for a direct connection of neural behavior with the subject's choice, without assumptions about the shape of the distribution of the neural responses [26]. A CP of 0.5 means that the observer would perform at chance. A CP significantly greater than 0.5 means that a higher firing rate predicts a behavior, while a CP significantly less than 0.5 indicates that a higher firing rate predicts the compared behavior. In order to determine whether CP values deviated significantly from chance we ran a permutation test (n = 2000). On each permutation, CP values were calculated from the firing rate on randomly assigned trial types. Experimental CP values were determined to be significant if they were outside the 95% CIs for that session. All CP values were calculated following a z-score correction of the firing rate [26].

### Subsampling of trials to identify likely movement-related “directional” units

In order to identify units whose responses were consistent with purely movement-direction-related activity attributable to motor preparation, execution, or sensory feedback (due for example to left or right whiskers and body contacting the chamber walls) due to response movements to the left or right, we wanted to count those units whose activity signaled right or left movements in both hit vs. CR trials and in FA vs. miss trials. Due to the high percent correct (77 SD 10) on average over the sessions used), there were often unequal numbers of trial types being compared, which affects CP calculations (Kang and Maunsell, 2012). Therefore in the analysis of movement-direction-related activity it was necessary to control for these different trial numbers, so that our statistical significance criterion would be equivalent for CPs for the same unit for different trial types – specifically the hit vs. CR CP and the FA vs. miss CP. In order to control for the potentially different numbers of trials of each type, when calculating CPs in the standard 2CD task we randomly selected trials such that the number of trials used to calculate the two CPs are the same within a session. For example, when calculating hit vs. CR CPs and FA vs. miss CPs for a given session, the totalnumber of hit and CR trials is the same as the total number of FA and miss trials for that session. If a session had fewer than 6 trials of a given type then it was excluded from the analysis. This resulted in exclusion of 25/44 sessions for this part of the analysis, with 2–6 sessions remaining from each of the five subjects. 20 random trial subsamples were calculated, and the data presented were averaged over these subsamples. This subsampling procedure likely reduced our statistical power but allowed us to treat the statistical significance criteria of hit vs. CR and FA vs. miss CPs as equivalent for each unit, in order to identify putative perception-related responses as described in the Results below.

### Firing rate analysis

In order to directly characterize the changes in firing rates that led to the observed choice probabilities, we compared the average baseline firing rate of units (calculated in two seconds prior to the initiation of a trial with a nosepoke) to the average firing rate in the two seconds following the signal time. If the average number of spikes in the two seconds following the stimulus was higher than the average number of spikes during two seconds prior to trial initiation, we say that this unit increases its firing rate in response to that stimulus.

We used a similar firing rate analysis in order to characterize units that appeared to ramp their firing rates up or down from baseline immediately preceding the signal/nonsignal time. We performed a two tailed t-test comparing the baseline firing rate in a 250 ms window 2 seconds prior to the stimulus to a 250 ms window immediately prior to the signal, across all trial types. Units that were found to significantly change in firing rate between these two bins were classified as ramp up or ramp down units.

## Results

### Movement- and perception-related activity

To characterize the frontal-parietal attention-detection network in rats we used multi-electrode arrays to record multi-unit spiking activity from medio-dorsal frontal and posterior parietal cortical neurons in 5 rats performing a two-choice auditory detection task (Figure 1A) in 19 separate recording sessions meeting our criterion for a minimum of six trials of each type (see Subsampling under Methods above). Figure 1B shows histograms of response latencies—the time between the signal onset (or equivalent post-nosepoke time for nonsignal trials) and the first lick for water at the left or right lickometer—from all these sessions. The average response latency was 1.2 seconds, and did not noticeably differ between the four trial types: hits, CRs, misses or FAs (Figure 1B). Animals performed at 77 SD 12% correct overall, with 70 SD 19% on signal trials and 80 SD 17% on nonsignal trials. This data set included a total of 320 multi-units in the frontal area and 652 from the parietal area; after discarding potentially duplicated units in different sessions recorded from the same rat (see Methods), our reduced dataset included 195 frontal and 349 parietal units. Results from this data set are shown in Table 1 for the full and reduced data sets respectively. As may be seen in Table 1 and as we detail below, removing the potentially duplicated units from our analysis did not substantially affect the results.

**Table 1:** Movement-direction- and perception-related activity.

	Frontal			Parietal		
Data set	CPU	Direction	PPU	CPU	Direction	PPU
**Full: 320 frontal, 652 parietal**	42/320 (13 SE 1%)	15/42 (36 SE 1%)	20/320 (8 SE 1%)	123/652 (19 SE 1%)	40/123 (33 SE 1%)	83/652 (13 SE 1%)
**Reduced: 195 frontal, 349 parietal**	31/195 (15 SE 1%)	13/31 (42 SE 2%)	18/195 (9 SE 2%)	80/349 (23 SE 1%)	31/80 (39 SE 1%)	49/349 (14 SE 1%)

For each data set, the CPU (Candidate Perceptual Unit) column shows the number (and percentage) of units in each area whose firing in a one-second post-stimulus window significantly differentiated hit from CR trials. The Direction column shows how many (what percentage) of the CPUs also significantly discriminated FA from miss trials, as well as signaling direction consistently across trials types, using matched numbers of trials in choice probability calculations for both trial-type pairs. The responses of these “directional” units were consistent with purely movement-related activity. Means and SEs were calculated using the variability across 19 sessions and 20 matched trial subsamples (see Methods). CPUs whose responses were not consistent with signaling movement direction were considered to be Putative Perceptual Units; these are tallied in the PPU column.

**Figure 2:**
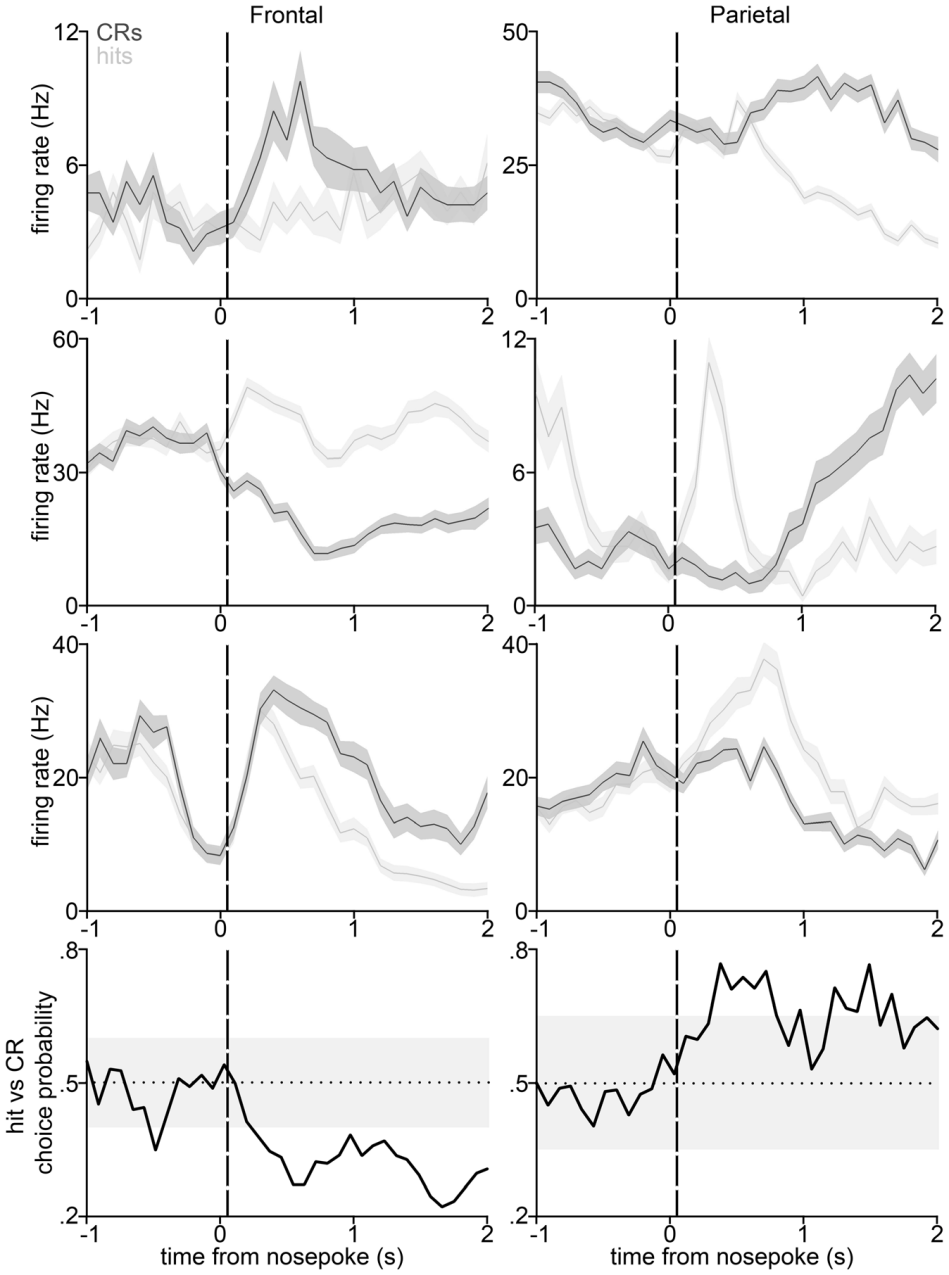
Example response patterns of three frontal (*left*) and three parietal (*right*) units on hit and CR trials. The *top three rows* show average firing rate over time (100 ms time bins, mean firing rate +/- SEM) for hit (*gray*) and correct rejection (*black*) trials. The *bottom row* shows choice probabilities over time for the bottom two units. *Horizontal dotted line* marks the CP expected by chance, and shading indicates the 95% confidence interval of the chance CP for that cell. *Vertical dashed line* indicates the target signal onset time, 10 ms after the nosepoke.

The top three rows of Figure 2 show spiking activity recorded from six units in frontal and parietal cortex whose responses discriminated hit from CR trials. These examples indicate the diversity of responses we observed, both in terms of temporal profile and in terms of which trial type induced higher firing rates.

To quantify neural correlates of task performance, we used a choice probability analysis [26] to measure the extent to which firing on individual trials predicted trial type. Figure 2 shows example CPs calculated in a 100 ms sliding window over time during a trial, based on firing rate differences between hit and CR trials. The CP time course quantifies the extent to which an ideal observer could predict which of the two trial types occurred based on firing rates at specific times during a trial. When it goes outside the shaded confidence interval we consider that unit's rate to carry significant information about whether the trial was a hit or a CR. In general, task-related spike rate differences between hits and CRs resulting in post-stimulus CPs statistically different from 0.5 (chance) could reflect two sources: perception of the target stimulus or different response movements including shifting the body left or right. As hits and CRs are both correct trials earning water reward, we expect no difference in reward processing between these trial types.

**Figure 3:**
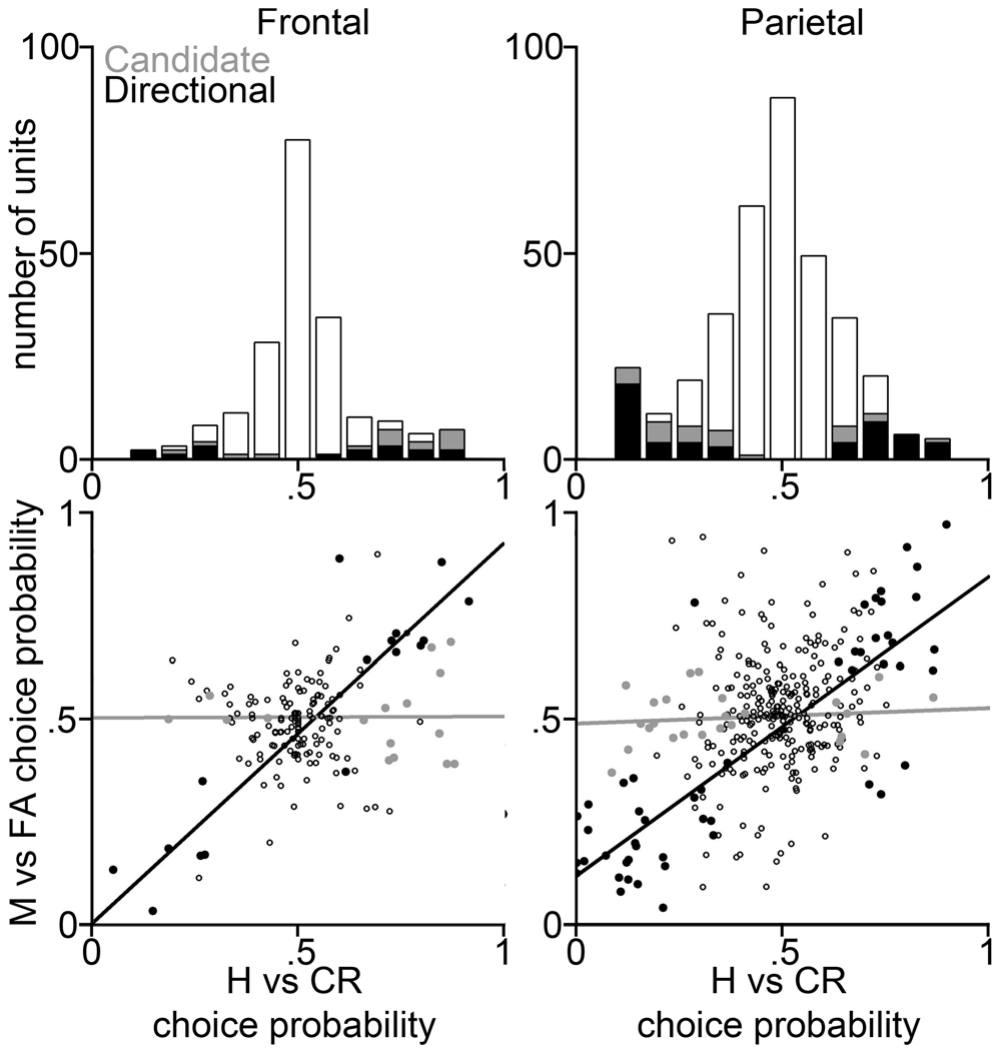
Choice probability distributions from example trial-subsamples of all 217 frontal (*left*) and all 252 parietal (*right*) units of our reduced data set. The *top panels* show CP histograms with the whole population shown in white, candidate perceptual units (CPUs, which discriminate hits from CRs) shown in gray, and directional units (see below) shown in black. The CPUs that are not directional (visible gray part of bar) are considered to be putative perceptual units (PPUs, see text). The *bottom panels* show Hit-CR vs. miss-FA CP magnitude comparisons for the frontal (*right*) and parietal (*left*) units. Lines represent best linear fit of miss-FA and hit-CR differentiating units (*black dots and line*) and hit-CR only differentiating units (*gray dots and line*). Units with non-significant CPs are shown as *unfilled circles*. Those black dots which have consistent rate modulations for right vs. left movements across trial types (i.e. horizontal and vertical coordinates either both >0.5 or both <0.5) are considered “directional” units which could be primarily signaling movement-direction rather than perception of the target tone. Those units tend to lie along the y = x line, consistent with the expectation that CPs should be approximately equal for direction-related units for hits vs. CRs as for FAs vs. misses; whereas the CPUs that do not also discriminate FAs from misses do not lie near y = x, consistent with a role in perception.

To focus on potential perceptual responses to the target tone, we calculated choice probabilities based on the first second of spiking activity following the signal for signal trials, and the first second following the beep latency for that session on nonsignal trials. To identify candidate perception-related unit responses we initially calculated choice probabilities based on a comparison of hit trials versus correct rejections (CRs). Over all units and subsamples, the average hit-CR CP for both areas did not differ from chance, with a mean CP of 0.52 SE 0.01 in frontal units, and 0.49 SE 0.01 in parietal units. Nevertheless, we found a substantial fraction of units in each area whose firing in the second after the time of the target tone discriminated hits from CRs—we term these “candidate perception units” (CPUs; Figure 3 top panels). Averaged over the 20 subsamples (see Methods), 15 SE 1% of units [31/195] in frontal cortex and 23 SE 1% [80/349 units] in parietal cortex had hit-CR CPs significantly different from 0.5 (Figure 3 top panels, light gray). The trend towards a greater fraction of CPUs in parietal cortex is not quite significant (p = 0.051, chi-squared test). Now, on hits the rat correctly reported the presence of the target tone by moving to the right lickometer for water, whereas on CRs the rat correctly reported the absence of the target tone by moving to the left for water. Thus firing-rate differences between hits and CRs could be due to modulation of a unit by left vs. right movement, or due to the unit's involvement in perceiving the target tone.

After identifying CPUs we determined which of these units could be signaling left vs. right movement direction by calculating CPs for FAs vs. misses. As in the hit-CR comparison, on FA and miss trials the rat moves in opposite directions, so we would expect the unit to carry significant information about both hits vs. CRs and FAs vs. misses, for units primarily registering motion direction. Importantly, in this analysis we matched the total trials numbers for the hit-CR and FA-miss CP calculations, so that the statistical significance criterion would be equivalent for the two CP calculations (see Methods). We found that 44 SE 2% [14/31] of CPUs in frontal cortex and 40 SE 1% [32/80] in parietal cortex could differentiate misses from FAs in addition to differentiating hits from CRs. Further analysis demonstrated that the large majority (98% and 95% in frontal and parietal cortex respectively) of CPUs that could significantly differentiate FAs from misses had CPs on the same side of 0.5, indicating consistent firing rate differences between both kinds of rightward trials (hits and FAs) and both kinds of leftwards trials (CRs and hits; see Figure 3 bottom panels). This response pattern is consistent with units signaling primarily movement direction, and so we count these units as direction-related in Table 1. We found that 42 SE 2% [13/31] of CPUs in frontal cortex and 39 SE 1% [31/80] in parietal cortex had “directional” responses in this sense. Although in principle some of these response modulations could actually represent perception-related activity, because they are consistent with movement-related signals we conservatively omit them from our estimate of the fraction of units whose responses are putatively related to the animal's perception. The linear fit to the units that discriminate both hits from CRs and FAs from misses (Figure 3 bottom panels, black fit line) lies close to y = x, consistent with the idea that CPs should be similar for the different trial types for units that are primarily signaling movement direction.

While movement-related responses would be expected to be similar for all movements in the same direction, perceptual responses could be weaker on incorrect trials (FAs and misses) than on correct trials (hits and CRs), or even inconsistent in terms of whether supposed reports of the same perception (e.g. hits and FAs) resulted in firing rate modulations in the same direction (e.g. up rather than down). Note also that the presence or absence of water reward is the same for both pairs of trial types that we are considering: reward for hits and CRs, no reward for FAs and misses. Therefore differences in reward between trial types cannot account for any of the significant CPs we have noted. For these reasons we consider CPUs whose responses are not directional to be putative perceptual units (PPUs). Approximately half of the CPUs that we found are not primarily involved in signaling movement direction, and are therefore considered to be putative perception units (PPU). Considering these PPUs as a fraction of all the units we recorded in each area, 18/195 frontal units and 49/439 parietal units of our reduced data set were PPUs (PPU column in Table 1). The proportions of PPUs in the two areas were not significantly different by a chi-squared test (p = 0.1). Table 1 also shows that the estimates of the fraction of PPUs in each area were comparable in the full and reduced data sets. Based on an analysis of PPU firing in the two seconds after the latency of the signal beep (see Methods), 49% of frontal PPUs and 55% of parietal PPUs signaled hits by decreased post-stimulus firing.

The response time distributions in Figure 1B provide relatively broad constraints on when detection of the beep takes place, and perceptual activity can last hundreds of milliseconds, so in the above to maximize our statistical power we used a wide 1 second window. However, it is likely that perception of the beep occurs often within about 250 ms of its onset. To examine this possibility we performed the same CP analysis using responses in the first 250 ms following the beep (rather than 1 second as in the analysis described above). As could be expected since we reduced our sampling window by a factor of 4, many fewer units were found to be PPUs using the shorter window, and there was more variation between subsamples: 6 SE 2% [11/195] of units in frontal cortex and 8 SE 1% [23/349] of units in parietal cortex (see Table 2).

**Table 2:** Movement and perception-related activity using a 250 ms time window.

	Frontal			Parietal		
Data set	CPU	Direction	PPU	CPU	Direction	PPU
**Full: 320 frontal, 652 parietal**	18/320 (6 SE 1%)	2/18 (14 SE 2%)	16/320 (5 SE 2%)	54/652 (8 SE 1%)	4/54 (8 SE 1%)	50/652 (8 SE 1%)
**Reduced: 195 frontal, 349 parietal**	13/195 (7 SE 1%)	2/13 (15 SE 2%)	11/195 (6 SE 2%)	32/349 (10 SE 1%)	3/32 (10 SE 2%)	29/349 (8 SE 1%)

Same conventions as in Table 1. Here, units were assessed as CPU, direction, and PPU using a 250 ms window following the signal, rather than a 1 second window.

**Figure 4:**
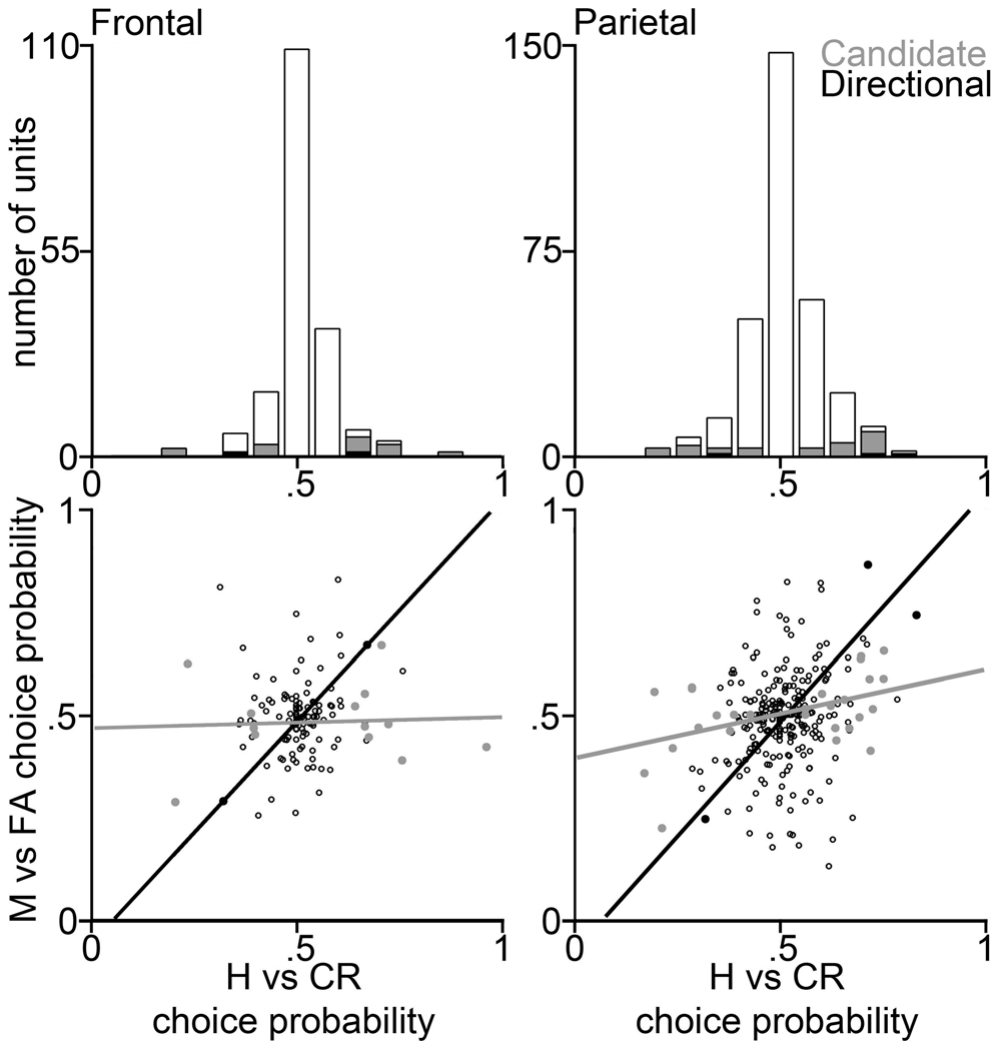
Choice probability distributions calculated in a 250 ms post-stimulus time bin from example trial-subsamples of all 195 frontal (*left*) and all 349 parietal (*right*) units of our reduced data set, using a 250 ms post-stimulus time bin. Conventions as in Figure 3.

Indeed the frontal estimate of the fraction of PPUs estimated using a 250 ms window is consistent with the fraction expected from chance fluctuations in the PSTHs. Still, as shown in Figure 4, the existence of some units with relatively large CPs suggests that at least some of the PPUs based on the 250 ms analysis are genuinely signaling perception rather than representing statistical fluctuations. Consistent with this interpretation, in the 250 ms analysis, a much smaller fraction of the CPUs were consistent with a motor-related function, as might be expected in the earlier time window (Figure 4).

### Rate modulation by task-irrelevant stimulus features

We also investigated the ability of units in these areas to differentiate tones based on task-irrelevant parameters, specifically the frequency or duration of the target tone. On average performance was 83 SE 11% correct for long tones, 75 SE 10 for brief tones, 74 SE 5 for high pitch tones, and 89 SE 6% correct for low pitch tones. For this analysis we did not require a minimal number of incorrect trials, so we did not need to omit sessions from the analysis on this basis. This data set therefore comprised 18 detection sessions recorded in five rats: 10 two-pitch sessions (4 rats, 163 frontal units and 167 parietal units in reduced data set after removing potential duplicates; 217 frontal and 231 parietal in full data set before removing potential duplicates), and 8 two-duration sessions (2 rats, 128 frontal and 176 parietal units after removing potential duplicates; 199 frontal and 285 parietal before removing potential duplicates). In these two-tone detection sessions the two tones were presented randomly in equal proportion. Rats were rewarded for responding to both tones in the same way.

Estimates of the fraction of CPUs in the different two-tone sessions were comparable to the results (Table 1) from the sessions with a minimum number of incorrect trials (n = 19) that we used in our analysis (above) aimed to identify motion-direction-related responses. For the 8 two-intensity sessions after discarding potentially repeated units, we found that 17% [22/128] of frontal units and 27% [47/176] of parietal units in were candidate perceptual units (CPUs), meaning they could significantly differentiate hits from correct rejections. Similarly, in the 10 two-pitch sessions 18% [29/163] of frontal and 22% [36/166] of parietal units were CPUs. These results may be compared to the proportions of CPUs listed for the reduced frontal and parietal data sets in Table 1.

**Figure 5:**
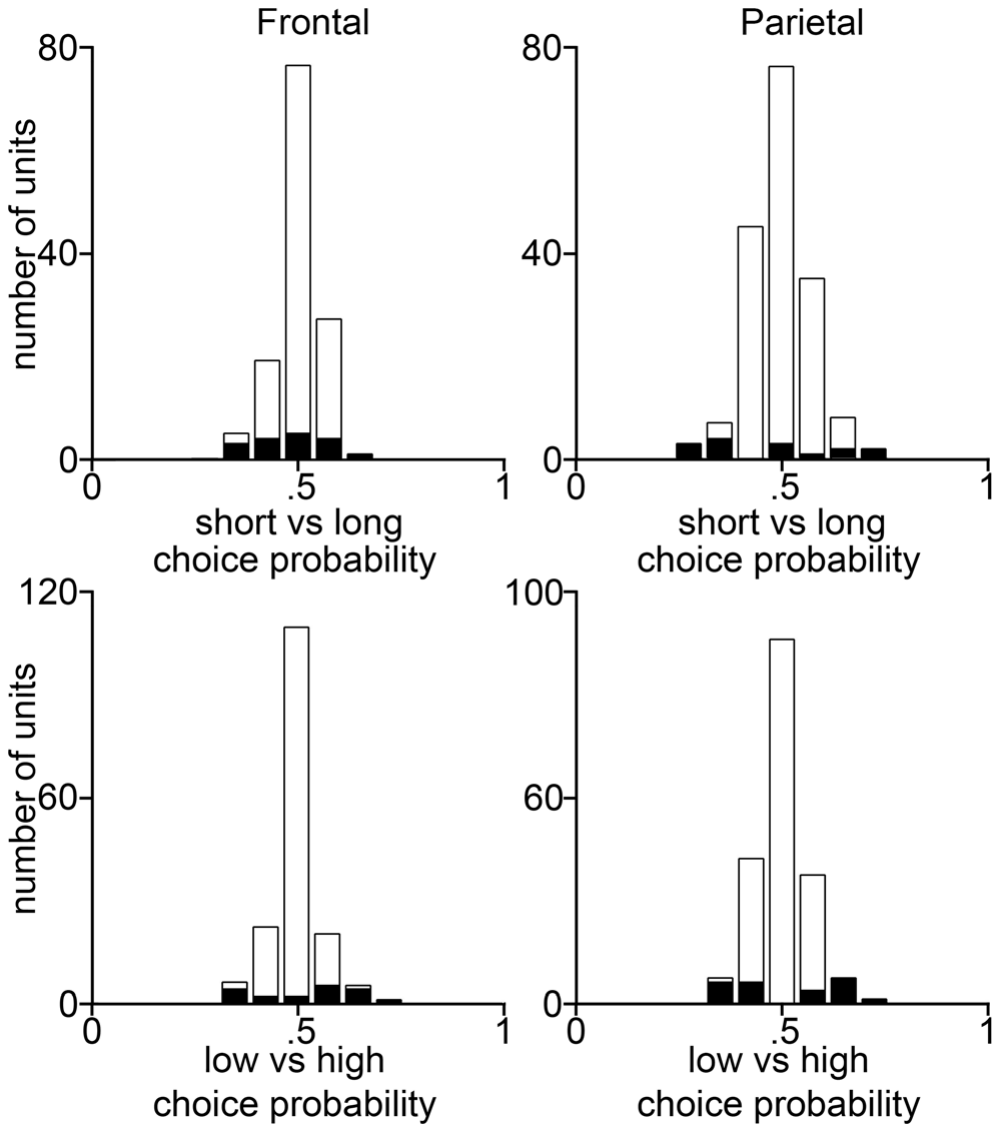
Task-irrelevant stimulus features. A small minority of units in frontal (*left*) and parietal (*right*) cortices significantly discriminated tones of different amplitudes (*top*) or tones of different pitches (*bottom*). 26/199 frontal and 31/285 parietal units significantly discriminated the tones of different amplitudes. 27/217 frontal and 28/231 parietal units significantly discriminated the tones of different frequencies. Data shown are from the reduced dataset (see Methods). Population shown in *white*; tone-differentiating units shown in *black*.

In order to test whether units in MFC or PPC were modulated by auditory stimulus parameters, we calculated CPs based on firing rate distributions on the two kinds of signal trials. (Although the name “choice probability” refers to neural information about a behavioral choice, for simplicity we use the same term in this non-behavioral case because the mathematical analysis is equivalent.) In this case, we compared high pitch tone trials to low pitch tone trials, and long-duration tone trials to brief tone trials. 13% [17/128] of frontal units and 9% [15/176] of parietal units could differentiate the two different duration stimuli (Table 3 and Figure 5, top panels). Of the duration-discriminating units, nearly equal numbers of units in each area preferred the long tone to the short tone: 7/17 frontal units were short tone-preferring, while the other 10/17 units preferred the long tone. 8/15 parietal units preferred the long tone; the other 7/15 preferred the short tone. Approximately one third of the duration-discriminating units were both CPUs and duration-discriminating units: 5/17 frontal units and 5/15 parietal units.

**Table 3:** Neural discrimination of task-irrelevant stimulus details.

Session Type		Frontal	Parietal
**Two-Duration: 2 rats, 8 sessions**	Full: 199 frontal, 285 parietal	26/199 (13%)	31/285 (11%)
	Reduced: 128 frontal, 176 parietal	17/128 (13%)	15/176 (9%)
**Two-Pitch: 4 rats, 10 sessions**	Full: 217 frontal, 231 parietal	27/217 (12%)	28/231 (12%)
	Reduced: 163 frontal, 167 parietal	18/163 (11%)	20/167 (12%)

The table shows the number (percentage) of units significantly distinguishing long and brief tones (two-duration sessions) or high and low pitches (two-pitch sessions), calculated in a 1 second post-stimulus window.

11% [18/163] of frontal units and 12% [20/167] of parietal units could differentiate the two different frequency stimuli (Fig 5, bottom panels). Of these frequency-discriminating units, more units in frontal cortex responded more strongly to the high tone than low tone: we observed 6/18 low-preferring and 12/18 high-preferring frontal units. We found 10/20 low-preferring and 10/20 high-preferring parietal units. Interestingly, only 3/18 frontal and 3/20 parietal pitch-discriminating units were also CPUs, suggesting that the task-irrelevant stimulus features are generally not processed by the same neurons that register the presence or absence of the target tone. Again, when we performed this analysis in a shortened, 250 ms time window, the percentages of units showing significantly discriminating responses dropped substantially (Table 4).

**Table 4:** Neural discrimination of task-irrelevant stimulus details in a 250 ms post-stimulus window.

Session Type		Frontal	Parietal
**Two-Duration: 2 rats, 8 sessions**	Full: 199 frontal, 285 parietal	10/199 (5%)	5/285 (2%)
	Reduced: 128 frontal, 176 parietal	1/128 (1%)	7/176 (4%)
**Two-Pitch: 4 rats, 10 sessions**	Full: 217 frontal, 231 parietal	7/217 (3%)	13/231 (5%)
	Reduced: 163 frontal, 167 parietal	3/163 (2%)	10/167 (6%)

The table shows the number (percentage) of units significantly distinguishing long and brief tones (two-duration sessions) or high and low pitches (two-pitch sessions), calculated in a 250 second post-stimulus window.

### Pre-stimulus activity

As can be seen in our example PSTHs (Figure 2), some units appeared to distinguish trial types prior to the onset-time of the signal tone (e.g. the second parietal unit down from the top in Figure 2). Differences in pre-stimulus activity between hit and CR trials could be due to anticipatory strategies favoring success on one or the other trial type, or such differences could reflect an animal's bias to respond one way or the other. To assess the incidence of such pre-stimulus trial-type information in the neural responses we recorded, we calculated CPs based on hit-CR firing rate differences prior to stimulus-time. The results are tabulated in Table 5 for a 1 second pre-stimulus window and in Table 6 for a 250 ms pre-stimulus window. Relatively few units showed such anticipatory responding, but the percentage is significantly different from the 5% expected from chance fluctuations in PSTHs in both frontal and parietal cortex, at least when using a 1 second analysis window.

**Table 5:** Trial type-differentiating activity in a 1 second pre-stimulus time window.

Data set	Frontal	Parietal
**Full: 320 frontal, 652 parietal**	24/320 (8 SE 1%)	54/652 (8 SD 1%)
**Reduced: 195 frontal, 349 parietal**	14/195 (7 SE 1%)	34/349 (10 SE 1%)

For each data set, the table shows the number (and percentage) of units in each area whose pre-stimulus firing significantly differentiated hit from CR trials. Means and SEs were calculated using the variability across 19 sessions and 20 matched trial subsamples (see Methods).

**Table 6:** Trial type-differentiating activity in a 250 ms pre-stimulus time window.

Data set	Frontal	Parietal
**Full: 320 frontal, 652 parietal**	19/320 (6 SE 1%)	44/652 (7 SD 1%)
**Reduced: 195 frontal, 349 parietal**	11/195 (6 SE 1%)	25/349 (8 SD 1%)

For each data set, the table shows the number (and percentage) of units in each area whose pre-stimulus firing significantly differentiated hit from CR trials. Means and SEs were calculated using the variability across 19 sessions and 20 matched trial subsamples (see Methods).

Another effect that is visible in the pre-stimulus time period for some units is ramping up or down of firing rate prior to the beep time. When we compared our CPU and directional unit populations to the population of units experiencing this ramp up or down effect (Table 7), we found substantial overlap. Across the entire population, 34% [109/320] of frontal units and 44% [287/652] of parietal units were found to have a ramp up or down effect, as measured by a t-test of baseline firing rate compared to the firing rate in the 250 ms preceding the beep (see Methods). Comparatively, 53 SE 2% of CPUs and 82 SE 1% of directional units in frontal cortex ramped their firing rates up or down immediately before the signal time. 55 SE 1% of CPUs and 61 SE 1% of directional units in parietal cortex showed this pattern. These results are collected in Table 7. Pre-stimulus ramp-down modulations were generally more common than ramp-ups in firing rate, though this trend did not reach statistical significance among the frontal units (chi-squared test: frontal p = 0.07, parietal p<0.001).

**Table 7:** Overlap of direction units and CPUs with ramp-up and ramp-down populations.

Unit type	Frontal Up	Frontal Down	Parietal Up	Parietal Down
**All units: 320 frontal, 652 parietal**	46/320 (14%)	63/320 (20%)	87/652(14%)	200/652 (31%)
**H-CR: 42 frontal, 123 parietal**	23/42 (27 SE 2%)	23/42 (26 SE 1%)	68/123 (20 SE 1%)	68/123 (35 SE 1%)
**Directional: 15 frontal, 40 parietal**	6/15 (43 SE 1%)	6/15 (39 SE 1%)	13/40 (32 SE 1%)	11/40 (29 SE 1%)

This table shows the number (percentage) of units classified as CPU and directional units that also showed ramp-up or ramp-down behavior preceding the signal. The first row reports the number of units across the entire population that have ramp up or ramp down behavior, defined as a significant (p<0.05) increase (“ramp-up”) or decrease (“ramp-down”) in firing rate in the 250ms preceding the signal, as compared with a 250ms baseline period 2 seconds prior to the signal. The second and third rows report the percent of CPU and directional units that show this behavior. Standard errors (SE) reflect variability over trial sub-sampling (see Methods); the first row shows no SEs because no trial sub-sampling was employed to identify the upwards and downwards pre-signal firing rate modulations.

## Discussion

### Spiking responses of a minority of MDFC and PPC units signal perception of a target tone

In order to characterize the spiking response to auditory stimuli of PPC and MDFC neurons in rats, we recorded simultaneously from both areas during an auditory two-choice detection task. 15–23% of units recorded in each area significantly discriminated correct response to signal trials (hits) from correct response to nonsignal trials (CRs), as measured by choice probability analysis. We considered these as candidate perceptual units (CPUs), since they reflected the presence or absence of the target tone. But about 40% (see Table 1) of the CPUs in each area also differentiated incorrect signal trials (misses) from incorrect nonsignal trials (FAs) consistent with motor or somatosensory activity related to different movement directions (left or right) in the different trial types. We conservatively interpreted these units as movement-related “directional” units, although their responses are also potentially consistent with a perceptual rather than movement-related role. That left a minority of about 9% of the total number of recorded units in frontal cortex, and 14% of units in parietal cortex whose responses were more consistent with a role in stimulus perception, which we considered as putative perceptual units (PPUs in Table 1). This value is likely to be an under-estimate, because (i) some of the responses considered as “directional” might actually be perceptual, and (ii) we may have missed some weaker but functionally distinct response modulations due to low numbers of trials in some cases. The response modulations we observed that correlate with the rat's perceptual report cannot be accounted for in terms of differences in reward processing in different trials, because we only compared trial types with equivalent rewards. Thus our results suggest that PPC and MDFC both contain a substantial fraction of neurons that signal perception of auditory signals, at least when the sounds are relevant to an ongoing attempt to get water. We tended to observe a greater fraction of PPUs in parietal than frontal cortex, but this tendency was not statistically significant.

Previous work has described frontal and parietal networks with activity related to visual and tactile perception in humans [5], [23], [27], [28], [29], non-human primates [30], [31], [32], and rats [23], [29], suggesting that these areas may participate in sensory perception and multi-sensory integration generally. It has also been indicated that parietal cortex has a role in the integration of visual and tactile stimuli in human imaging [33], transcranial magnetic stimulation [34], and non-human primate electrophysiology studies [35]. Despite evidence that PPC and prefrontal cortex (PFC) may be involved with the integration of auditory and visual information as well [36], very little has been reported regarding either area's response to auditory stimuli. In primates and humans [36] as well as rats [21] PPC processes auditory as well as visual and tactile inputs, but little has been done to establish spiking neural correlates of auditory perception in frontal or parietal cortex outside of the dedicated single-sensory-modality areas [20]. Thus, to our knowledge, our results are the first to identify spiking correlates of auditory perception in the MDFC and PPC of rats.

Still, our results are broadly consistent with previous recordings in these areas in rats. Narayanan and Laubach [17] recorded in MDFC while rats performed a delayed response task in which the behavioral response was triggered by an auditory tone, and reported that 14% of recorded neurons there signaled the tone, comparable to our estimate of about 9% of MDFC units manifesting auditory perceptual responses in our experimental context. Similarly, Broussard et al [22] found that 25% of PPC neurons in rats performing a visual detection task signaled the presence of the visual target. However, in contrast to these studies, where the presence of the trigger or target stimulus was generally signaled by firing rate increases, in our data firing rate increases and decreases were about equally likely to signal the presence of the target tone. It is possible that this discrepancy may be explained in terms of the different stimulus modality in the Broussard et al study [22], but it is unclear how the different task demands in our experiments as compared to those of Narayanan and Laubach [17] could account for the difference in response profiles that we observed.

### A smaller, mostly non-overlapping minority of units in MDFC and PPC discriminates task-irrelevant auditory stimulus features

In the primate and rodent sensory physiology literature, a distinction is sometimes drawn between “perceptual” neural responses reflecting an animal's behavioral report about a sensory stimulus, as distinguished from more purely “sensory” responses reflecting details about the stimulus that might not be relevant to the animal's current goals. In an investigation of the neural correlates of tactile perception in monkeys, the firing rates of frontal neurons tended to correspond to the animal's perception of a tactile stimulus, while parietal neurons in sensory areas S1 and S2 tend to instead reflect task-irrelevant stimulus properties such as intensity, independently of the animal's perceptual report [24]. In contrast, firing of PPC neurons in rats appears to correlate with perceptual report rather than with task-irrelevant features of a visual stimulus [22]. The parietal sensory areas sampled in the monkey study are not directly comparable to the posterior parietal area studied in the rat paper (or in the present study), but these observations nevertheless raise the question: at what processing stages are sensory responses reflecting task-irrelevant sensory features transformed into the responses reflecting the task-relevant sensory feature(s) and the perceptual report?

We were therefore interested to test whether frontal neurons display more “perceptual” responding and parietal neurons display more “sensory” responses in the context of our auditory detection task. To this end, we recorded during detection sessions involving two distinct but equally rewarded target tones, differing either in terms of duration or tone pitch (in separate sessions). Consistent with a study of PPC responses during visual detection in rats [22], which found predominantly perceptual responses that did not reflect the task-irrelevant duration of the visual target, we found very few units in PPC or MDFC whose firing rates distinguished long-duration from brief or high from low frequency tones (Tables 3 & 4). Those few units that did discriminate task-irrelevant tone parameters were rarely candidate perceptual units (CPUs, which discriminate hits from CRs), supporting that sensory representations in rats are implemented in mostly separate populations of neurons from those implementing the perceptual representations, as was found in the study of tactile perception in monkeys [30], [31]. The firing rate modulations that did discriminate task-irrelevant stimulus features might provide a substrate for these cortical areas to build enhanced representations on, should these features of the sound become behaviorally important in the future.

De Lafuente and Romo (2006) suggested that neural correlates of tactile perception in the monkey build up progressively along the processing stream from parietal sensory areas to areas in frontal cortex. However, as we noted above, that study did not sample PPC, so it is not known whether PPC neurons in the monkey exhibit the perceptual responses characteristic of the frontal neurons in that study. The perceptual responses we observed in the rat were represented relatively sparsely in MDFC and PPC, with a tendency towards greater numbers in PPC. Thus, while our study establishes analogous auditory perceptual spiking responses in rat frontal and parietal cortices as have been observed in the tactile modality in primate cortex, future work will be needed to determine whether the frontal-parietal distribution of perceptual responses is similar in the rat and primate brain. Similarly, it is desirable to corroborate the present results with recordings near the animal's detection threshold [37], [38] and with behavioral responses reversed in different blocks of trials to definitively control for movement-related activity.

## Conclusion

Despite impressive advances made in neuroscience within the past century, the neural mechanisms that underlie conscious perception are still not well understood [39]. While progress has been made in identifying neural correlates of conscious perception in humans [7], [39], [40], understanding the relevant neural mechanisms will likely require invasive experiments in non-human animals. Moreover, progress understanding neural mechanisms of cognition including perception itself will depend on the ability to manipulate specific neural players, rather than merely recording their activity. Manipulations such as optogenetic stimulation of genetically defined cell-types [41], [42] will be critical for testing the causal role of different brain areas and cell types in perception, but these techniques are usually developed earlier in rodent than primate animal models, and it is generally more feasible to apply the techniques in greater numbers of rodent subjects than primates. These considerations motivate the establishment of rodent models of cognition [20].

Our identification of spiking responses in rat frontal and parietal cortex that signal the rat's perception of an auditory target establishes that auditory perception is represented among MDFC and PPC neurons, by a neural population that does not substantially overlap with neurons representing task-irrelevant sensory features. These results set the stage for experiments to test the causal role of MDFC and PPC neurons in perception.
